# Bilateral Facial Nerve Palsy Revealing Diffuse Large B-Cell Lymphoma With Leptomeningeal Involvement

**DOI:** 10.7759/cureus.86828

**Published:** 2025-06-26

**Authors:** Al Motasim Bella Abu Laban, Waleed Muhammad, Nabih Hanbali

**Affiliations:** 1 Medicine and Surgery, Royal Surrey County Hospital, Guildford, GBR; 2 Acute Internal Medicine, Royal Surrey County Hospital, Guildford, GBR; 3 Radiology, Royal Surrey County Hospital, Guildford, GBR

**Keywords:** bilateral facial palsy, central nervous system lymphoma, cranial neuropathy, diffuse large b-cell lymphoma, leptomeningeal involvement, neuroimaging

## Abstract

We present a diagnostically challenging case of a 54-year-old woman who developed progressive bilateral lower motor neuron (LMN) facial nerve palsy, later accompanied by lower limb weakness and impaired dexterity. The initial presentation was misattributed to idiopathic Bell’s palsy, which delayed recognition of a more serious underlying condition. Neuroimaging revealed enhancement of multiple cranial nerves and diffuse leptomeningeal involvement. Cerebrospinal fluid (CSF) analysis demonstrated lymphocytic pleocytosis, elevated protein levels, low glucose, and increased lactate dehydrogenase (LDH), findings suggestive of a malignant infiltrative process. Additional imaging of the spine and plexus showed diffuse marrow signal abnormalities, cauda equina enhancement, and a right C5 nerve root mass with bony erosion. A diagnosis of stage IVA diffuse large B-cell lymphoma (DLBCL) with central nervous system (CNS) and leptomeningeal involvement was established during admission. The patient was referred to hematology, and a multidisciplinary team recommended systemic chemotherapy with methotrexate, cytarabine, thiotepa, and rituximab (MATRix) or rituximab, ifosfamide, carboplatin, and etoposide (RICE), followed by autologous stem cell transplantation for consolidation. This case highlights a rare presentation of CNS lymphoma and underscores the importance of early consideration of malignancy in patients presenting with progressive cranial neuropathies and polyradiculopathy, particularly in the absence of constitutional or systemic symptoms. Timely neuroimaging and CSF analysis were instrumental in achieving an accurate diagnosis, allowing for early initiation of appropriate therapy in this aggressive disease.

## Introduction

Diffuse large B-cell lymphoma (DLBCL) is the most common subtype of non-Hodgkin lymphoma (NHL), accounting for approximately 30%-40% of adult cases worldwide [1]. It typically presents with rapidly enlarging lymphadenopathy, constitutional symptoms such as weight loss or fever, and extranodal involvement affecting sites like the gastrointestinal tract, bone, or skin. While central nervous system (CNS) dissemination is a recognized complication, it more commonly occurs as a relapse in high-risk cases rather than at initial diagnosis [2].

Leptomeningeal involvement, referring to infiltration of the pia and arachnoid mater, the thin membranes covering the brain and spinal cord, is a rare and diagnostically challenging manifestation. It often presents with a range of neurological symptoms, necessitating a high index of clinical suspicion and confirmation via advanced neuroimaging and cerebrospinal fluid (CSF) analysis [3].

Cranial neuropathies, involving dysfunction of one or more cranial nerves, are rare presenting features of lymphoma. Lower motor neuron (LMN) facial nerve palsy results in weakness of all muscles on one side of the face due to damage occurring after the nerve exits the brainstem. Bilateral LMN facial palsy, affecting both sides of the face, is extremely uncommon in lymphoma and may be misdiagnosed as more benign conditions such as idiopathic Bell’s palsy or Guillain-Barré syndrome [4]. In the absence of systemic symptoms or palpable lymphadenopathy, hematological malignancy may not be considered early, leading to potentially significant diagnostic delays. This is clinically relevant, as early diagnosis and initiation of treatment are crucial in improving outcomes in aggressive diseases like CNS lymphoma [5].

This case report highlights a rare presentation of DLBCL with extensive leptomeningeal and CNS involvement, initially manifesting solely as bilateral LMN facial palsy and progressive polyradiculopathy, a condition characterized by widespread nerve root involvement, often resulting in limb weakness or sensory deficits. The diagnosis was established through a combination of neuroimaging and CSF analysis, allowing for timely referral and initiation of appropriate hematological treatment. This case underscores the importance of including lymphoma in the differential diagnosis of atypical cranial neuropathies, particularly when radiological and biochemical findings suggest an infiltrative process [6,7].

## Case presentation

A 54-year-old woman with no significant past medical history presented with progressive neurological symptoms over several weeks. She initially developed left-sided LMN facial nerve palsy, which was clinically diagnosed as Bell’s palsy and managed conservatively. However, her condition worsened, with the subsequent onset of contralateral facial weakness, progressive bilateral lower limb weakness, and impaired hand coordination. She denied sensory disturbances, bowel or bladder dysfunction, and systemic symptoms such as fever or weight loss.

On neurological examination, she exhibited bilateral LMN facial palsy, reduced muscle strength in the lower limbs (MRC grade 4/5), and generalized areflexia. Apart from facial nerve involvement, no other cranial nerve deficits were noted. There were no signs of meningeal irritation or cerebellar dysfunction. The clinical presentation raised concern for an infiltrative or autoimmune neuropathy, with differential diagnoses including Guillain-Barré syndrome, sarcoidosis, and CNS lymphoma.

**Figure 1:**
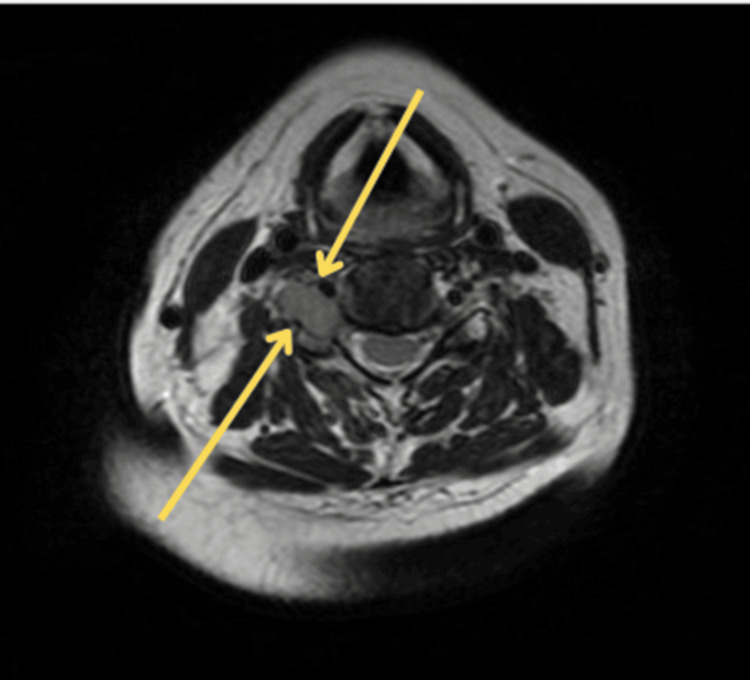
Enhancing right C5 nerve root on MRI Axial MRI of the cervical spine demonstrating a well-defined enhancing lesion involving the right C5 nerve root (yellow arrows), suggestive of lymphomatous infiltration.

Magnetic resonance imaging (MRI) of the brain demonstrated bilateral enhancement of cranial nerves VII and VIII, along with thickened meninges. Spinal imaging revealed an enhancing lesion at the right C5 nerve root with adjacent bony involvement, as well as patchy enhancement of the cauda equina (Figure 1).

**Figure 2:**
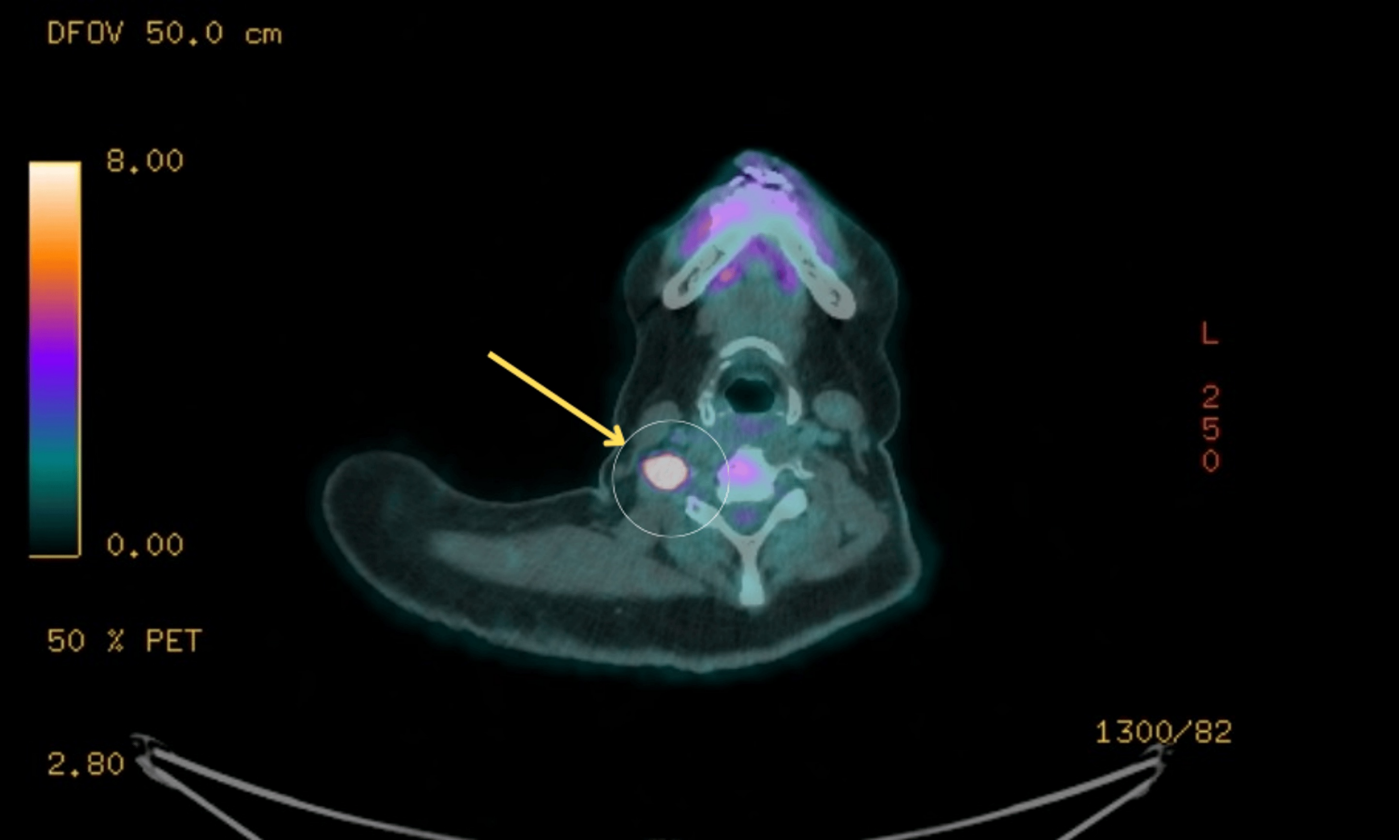
FDG-avid lesion at the right C5 nerve root on PET-CT Axial PET-CT image demonstrating intense fluorodeoxyglucose (FDG) uptake at the right C5 nerve root (yellow arrows). This hypermetabolic focus is suggestive of active lymphomatous infiltration of the cervical nerve root, supporting a diagnosis of central nervous system lymphoma.

**Figure 3:**
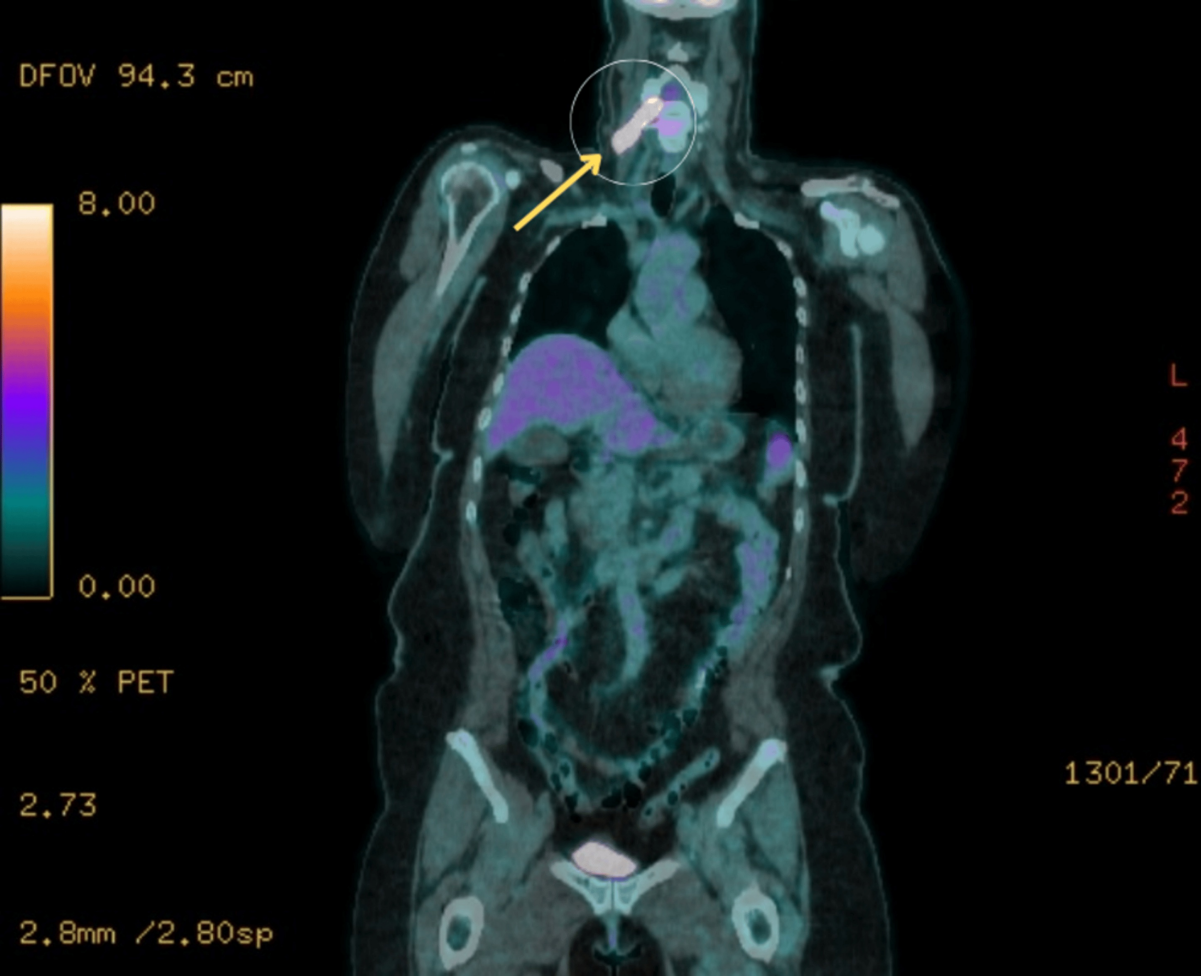
Diffuse FDG uptake along the spinal nerve roots and marrow on coronal PET-CT Coronal PET-CT image showing widespread fluorodeoxyglucose (FDG) uptake along the spinal nerve roots (yellow arrows). The diffuse metabolic activity is consistent with central nervous system involvement by lymphoma. No FDG-avid lymphadenopathy or visceral organ involvement is observed, indicating a non-nodal, extranodal pattern of disease distribution.

Positron emission tomography-computed tomography (PET-CT) revealed a corresponding fluorodeoxyglucose (FDG)-avid focus at the C5 nerve root (Figure 2), along with diffuse FDG uptake throughout the bone marrow and spinal nerve roots, in the absence of significant lymphadenopathy or visceral organ involvement (Figure 3). These radiological findings were highly suggestive of lymphomatous infiltration with leptomeningeal spread.

CSF analysis revealed lymphocytic pleocytosis, elevated protein concentration, reduced glucose levels, and markedly elevated lactate dehydrogenase (LDH), as summarized in Table 1. Cytological examination identified atypical lymphoid cells, consistent with malignant infiltration. A subsequent bone marrow biopsy confirmed the diagnosis of DLBCL with CNS and leptomeningeal involvement. Notably, no mass lesions or nodal enlargement was seen on imaging, further emphasizing the atypical and extranodal presentation of the disease.

**Table 1:** Cerebrospinal fluid (CSF) analysis suggestive of leptomeningeal involvement

Parameter	Result	Normal range	Interpretation
White cells	125/μL	0-5/μL	Lymphocytic pleocytosis
Protein	2.6 g/L	0.15-0.45 g/L	Elevated
Glucose	1.5 mmol/L	2.2-3.9 mmol/L (CSF)	Reduced (serum glucose: 5.4 mmol/L)
LDH	168 U/L	0-40 U/L	Markedly elevated
Cytology	Atypical lymphoid cells	–	Malignant infiltration consistent with lymphoma

The patient commenced high-dose CNS-directed chemotherapy following a multidisciplinary team discussion. During the initial phase of the treatment, she developed pancytopenia, attributed to chemotherapy-induced bone marrow suppression. Supportive measures were initiated, including blood transfusions and close monitoring for infections. She remains under active hematological management, with ongoing neurological follow-up to monitor her response to therapy.

## Discussion

DLBCL remains the most common subtype of non-Hodgkin lymphoma (NHL), characterized by aggressive clinical behavior and diverse presentations [1]. While DLBCL typically presents with nodal enlargement or systemic symptoms, CNS involvement is relatively uncommon and usually occurs in the context of relapse or advanced disease [2]. Leptomeningeal infiltration, in particular, represents a rare and diagnostically challenging manifestation [3].

In the present case, the patient exhibited progressive bilateral LMN facial palsy, followed by lower limb weakness and areflexia. These clinical features initially raised suspicion for inflammatory neuropathies, such as Guillain-Barré syndrome or neurosarcoidosis. However, MRI revealed a right-sided C5 nerve root lesion with associated meningeal thickening, and PET-CT showed diffuse FDG uptake along the spinal nerve roots and bone marrow, without evidence of nodal or visceral involvement, findings that strongly suggested an infiltrative hematological malignancy.

Although facial nerve involvement has been documented in hematologic malignancies, bilateral LMN facial palsy remains exceedingly rare in lymphoma and is more frequently associated with viral or autoimmune etiologies [4]. The absence of systemic lymphadenopathy or organomegaly in this patient contributed to a diagnostic delay, as seen in other reported cases of leptomeningeal lymphomatosis presenting with cranial neuropathies or polyradiculopathy [8,9]. These observations emphasize the importance of maintaining a high index of suspicion for lymphoma, even when classical systemic features are lacking.

CSF analysis was pivotal in establishing the diagnosis. The presence of lymphocytic pleocytosis, elevated protein, reduced glucose, and elevated LDH, coupled with cytological identification of atypical lymphoid cells, was consistent with leptomeningeal involvement by lymphoma. When interpreted alongside radiologic findings, these results provided the foundation for prompt initiation of lymphoma-directed therapy.

Management of CNS lymphoma generally involves high-dose methotrexate-based regimens, such as MATRix, while salvage regimens like RICE may be used in selected cases [6]. In this case, the patient responded favorably to initial chemotherapy, demonstrating early neurological improvement. Where feasible, consolidation with autologous stem cell transplantation (ASCT) may offer improved long-term outcomes [7].

Despite advances in therapy, CNS involvement in lymphoma continues to be associated with a guarded prognosis. This case underscores the need for early recognition and timely multidisciplinary evaluation in patients presenting with progressive cranial neuropathies or spinal symptoms. Including leptomeningeal lymphoma in the differential diagnosis, even in the absence of systemic disease, may facilitate earlier diagnosis and intervention, ultimately improving clinical outcomes.

## Conclusions

This case highlights a rare presentation of DLBCL with primary leptomeningeal involvement, initially manifesting as progressive bilateral LMN facial palsy and polyradiculopathy. The absence of systemic symptoms and lymphadenopathy contributed to a delayed diagnosis, underscoring the importance of early neuroimaging and CSF analysis in patients presenting with atypical neurological features. Cytological identification of malignant lymphoid cells, along with correlative PET-CT findings, was instrumental in confirming the diagnosis. Timely initiation of CNS-directed chemotherapy resulted in early neurological improvement. This case reinforces the need to maintain a high index of suspicion for leptomeningeal lymphoma in patients with multifocal cranial or spinal nerve involvement, even in the absence of systemic disease, and highlights the critical role of a multidisciplinary approach in achieving prompt diagnosis and management.
